# Bilateral multifocal inverted papilloma with osseous metaplasia of the sinonasal cavity^☆^^☆☆^

**DOI:** 10.1016/j.bjorl.2016.06.012

**Published:** 2016-07-30

**Authors:** Lokman Uzun, Seyma Ozkanli, M. Tayyar Kalcioglu, Numan Kokten, Cigdem Kafkasli

**Affiliations:** a Istanbul Medeniyet University Medical Faculty, Goztepe Training and Research Hospital, Department of Otorhinolaryngology, Istanbul, Turkey; b Istanbul Medeniyet University Medical Faculty, Goztepe Training and Research Hospital, Department of Pathology, Istanbul, Turkey

## Introduction

Metaplasia is the reversible replacement of one differentiated cell type or tissue with another mature differentiated cell type or tissue. The change from one cell type to another may be part of a normal maturation process or caused by an abnormal stimulus.^1^ An inverted papilloma is a benign epithelial tumor composed of well-differentiated columnar or ciliated respiratory epithelium having variable squamous differentiation; it has a high recurrence rate and a squamous cell carcinoma association. Although bony metaplasia has frequently been described in polyps of the gastrointestinal tract, it is rarely encountered in the head and neck region.^1^, ^2^ In the head and neck region, lipomas are the most common lesions with osseous metaplasia; these are known as osteolipomas.^3^ Osseous metaplasia is rarely encountered in nasal polyp cases and has not been reported in inverted papilloma.

## Case report

**Figure 1:**
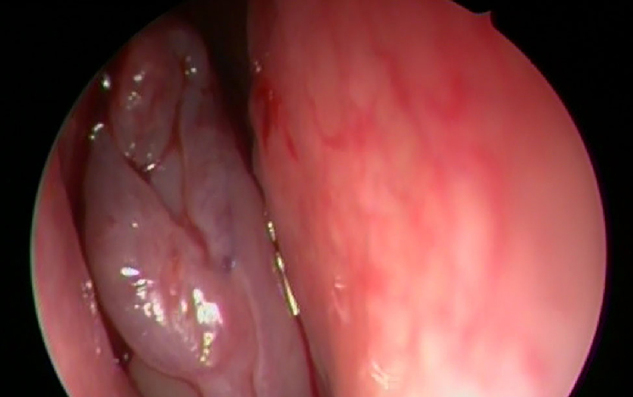
Endoscopic view of the sinonasal mass.

**Figure 2:**
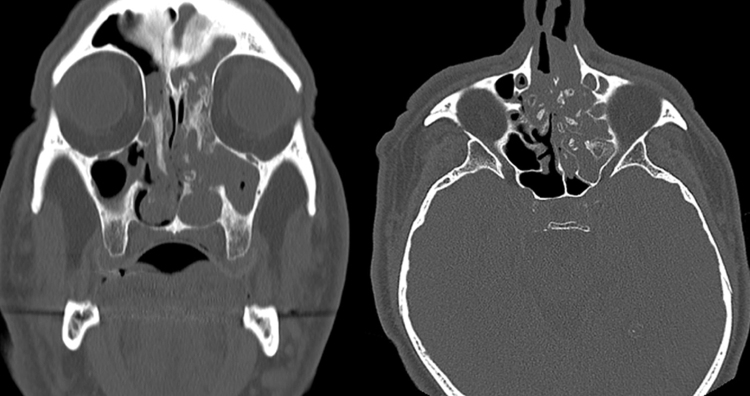
Bilateral multifocal osseous mass with soft tissue lesions axial and coronal CT images.

**Figure 3:**
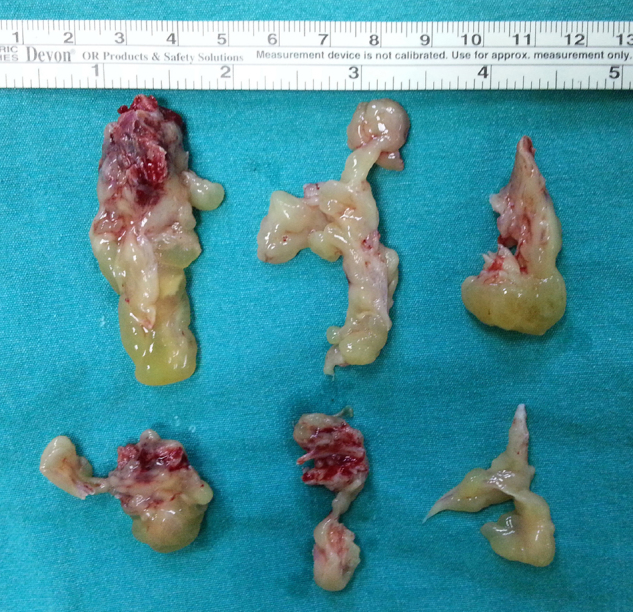
Removed pathological tissues at the operation. They include separate osseous mass attached to the inverted papilloma tissues.

**Figure 4:**
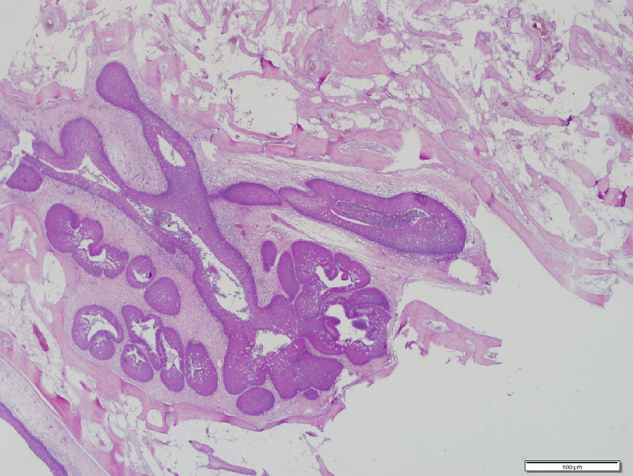
Nonkeratinized squamous epithelium shows inverted development. Numerous mucous cells in the epithelium and epithelium adjacent to the osseous metaplasia, which contains bone marrow (H&E, original magnification 40×).

A 68-year-old patient with bilateral nasal obstruction for 10 months was admitted to our clinic. He did not disclose any other nasal symptoms, such as headache, epistaxis, or rhinorrhea. The patient had no history of previous nasal diseases or surgery. Physical examination, including nasal endoscopy, revealed a bilateral multilobulated nasal mass filling both nasal cavities (Fig. 1). An imaging study was carried out using Computed Tomography (CT) of the paranasal sinuses. This showed a soft tissue mass with osseous foci in both nasal cavities (Fig. 2). Endoscopic surgery was performed, and all pathologic tissues were removed (Fig. 3). Histopathologic examination revealed an inverted papilloma with osseous metaplasia and inflammatory polyps for both sites (Fig. 4). No postoperative complication occurred during the follow-up period. Six months after surgery, endoscopic evaluation was performed and noted no pathologic view.

## Discussion

Metaplasia is the conversion of one type of tissue into a tissue different from its lineage.^1^ Genetic reprogramming of epithelial stem cells or undifferentiated mesenchymal cells is thought to cause the metaplasia.^3^ Metaplasia can occur in epithelial tissue or connective tissue.^2^ As a representative of connective tissue metaplasia, osseous metaplasia, which is an extremely rare pathology in the nasal cavity, is usually accompanied by nasal polyposis.^4^ Osseous metaplasia is also referred to as metaplastic ossification or ectopic bone formation.^3^, ^4^ The etiology of osseous metaplasia is unknown; the differentiation of an adult cell type to an osteoblast is assumed.^3^ The presence of a pluripotential cell or dedifferentiation of a cell into a pluripotential cell in tissues is assumed to cause ectopic bone formation.^5^ Lipomas are the most common lesions with osseous metaplasia in the head and neck regions. Previous surgery has been suggested as a trigger for the development of osseous metaplasia.^4^ However, in agreement with most reported cases of osseous metaplasia of the sinonasal region, our case had no history of sinonasal surgery.^1^, ^2^, ^3^, ^4^, ^5^ Calcification of nasal polyps due to hypercalcemia have been reported,^4^ but the serum calcium level of our case was normal. Hamartomas are tissue development errors and consist of abnormal mixture of focal tissues.^5^ Calcification can be seen in hamartomas, but our case did not have a hamartoma or congenital lesion. Also fungus ball in the paranasal sinuses may cause calcification. These lesions can be diagnosed easily with computed tomography.

Although focal hyperostosis is an expected finding,^6^ this is the first report of an osseous metaplasia with inverted papilloma. Lee et al.^6^ studied focal hyperostosis on a CT study of sinonasal inverted papillomas as a predictor of tumor origin. Eccentric bone thickening and sclerosis were defined as focal hyperostosis for only a limited portion of the wall of the paranasal sinus, and focal hyperostosis on CT scans was argued for detecting the origin of inverted papilloma preoperatively via CT.^6^ Inverted papilloma is important for its malignancy potential and high recurrence rate.^3^, ^6^, ^7^ Here, we presented a case with bilateral, multifocal, inverted papilloma with osseous metaplasia. Although inverted papillomas commonly are presented unilaterally and originate from the lateral nasal wall or the middle meatus, our case was bilaterally presented. After surgical treatment, histopathologic examination revealed a bilateral inverted papilloma with osseous metaplasia.

The differential diagnosis of osseous sinonasal masses include especially hyperostosis, also bone sequestration, long-lasting fungal sinusitis, metaplasia, and inverted papilloma.^1^, ^4^ Inverted papilloma must be ruled out first when faced with an osseous sinonasal mass because of its malignancy potential, high recurrence rate, and the locally aggressive character of the tumor. Hyperostosis is a benign lesion that occupies the internal surface of the bone it is seen on and it grows exuberantly. Histologically, lamellar bone is seen on the large regions and the remodeling of cranial region can be seen. In our case, osseous regions have no relation with the bone both radiologicaly and pathologically. Osseous metaplasia is the presence of the bone in the soft tissue. Although its pathogenesis is not clearly known, the most accepted theory is that mesenchymal pluripotent cells differentiate to osteoblast progenitors by the effect of bone morphogenetic proteins (BMPs) and transforming growth factor B1 (TGF-B1) and then secretion of the osteoblasts and bone matrix occurs with the osteogenic signaling.^8^ In our case, as there is a mature trabecular bone tissue unconnected with the bone tissue in the middle of the polyp under the respiratory epithelia, we accepted it as an osseous metaplasia.

## Conclusion

To the best of our knowledge, this is the first reported case of inverted papilloma with osseous metaplasia. Our findings indicate that inverted papilloma may be an unusual presentation of osseous sinonasal masses.

## Ethical approval

The authors assert that procedures contributing to this work comply with the ethical standards of relevant national and with the Helsinki Declaration 1975, as revised 2008.

## Conflicts of interest

The authors declare no conflicts of interest.
